# The *Salmonella* Paratyphi A O-Antigen Glycoconjugate Vaccine Is Able to Induce Antibodies with Bactericidal Activity Against a Panel of Clinical Isolates

**DOI:** 10.3390/vaccines13020122

**Published:** 2025-01-25

**Authors:** Marika Pinto, Salvatore Durante, Martina Carducci, Luisa Massai, Renzo Alfini, Elli Mylona, Abhilasha Karkey, Stephen Baker, Francesca Micoli, Carlo Giannelli, Omar Rossi, Simona Rondini

**Affiliations:** 1 GSK Vaccines Institute for Global Health (GVGH), via Fiorentina 1, 53100 Siena, Italy; marika.x.pinto@gsk.com (M.P.); salvatore.x.durante@gsk.com (S.D.); martina.x.carducci@gsk.com (M.C.); luisa.x.massai@gsk.com (L.M.); renzo.x.alfini@gsk.com (R.A.); francesca.x.micoli@gsk.com (F.M.); carlo.x.giannelli@gsk.com (C.G.); omar.x.rossi@gsk.com (O.R.); 2 GSK, via Fiorentina 1, 53100 Siena, Italy; 3 Cambridge Institute of Therapeutic Immunology and Infectious Disease, Department of Medicine, University of Cambridge, Cambridge CB2 0AW, UK; eem55@cam.ac.uk (E.M.); sgb47@cam.ac.uk (S.B.); 4 Oxford University Clinical Research Unit, Patan Academy of Health Sciences, P.O. Box 26500, Kathmandu 44700, Nepal; akarkey@oucru.org

**Keywords:** *Salmonella* Paratyphi A, vaccine, O-antigen, glycoconjugate, O-acetylation, Luminescence-based serum bactericidal assay

## Abstract

Background: Typhoid and paratyphoid fevers represent a global health burden, especially in Southern Asia, exacerbated by the increase in antimicrobial resistance. While vaccines against *Salmonella* Typhi have been successfully introduced, a vaccine against *S.* Paratyphi A is not available, yet. Efforts to develop an effective vaccine targeting both *Salmonella* serovars are currently ongoing. GVGH is developing a bivalent vaccine constituted by the Vi-CRM_197_ typhoid conjugate vaccine (TCV), and the *Salmonella* Paratyphi A O-antigen (O:2), also conjugated to the CRM_197_ carrier protein (O:2-CRM_197_). In this work we have characterized a panel of *S.* Paratyphi A clinical isolates from endemic regions, differing in terms of their O:2 structural features. Methods: Rabbits were immunized with the *S.* Paratyphi A component of the vaccine candidate and the resulting sera were tested for their ability to bind and kill the isolates using flow cytometry and luminescence-based serum bactericidal assay (L-SBA). Results: The O:2-CRM_197_ glycoconjugate induced a functional immune response in rabbits, effectively binding and killing a diverse panel of clinical isolates. The sera demonstrated bactericidal activity independent of the O:2 structural variations, including differences in O-acetylation and glucosylation levels. Additionally, the study found that the O:2-CRM_197_ conjugate’s adsorption to Alhydrogel did not significantly impact its immunogenicity or bactericidal efficacy. Conclusions: The O:2-CRM_197_ component of the bivalent vaccine candidate shows promise in providing broad protection against *S*. Paratyphi A isolates, regardless of their O-antigen structural variations. The ongoing clinical studies on human sera are expected to confirm these results.

## 1. Introduction

Enteric fever is a significant health issue, particularly in those countries where access to clean water and sanitation is inadequate [1,2]. It is caused by *Salmonella enterica* serovars Typhi and Paratyphi A, human-restricted pathogens transmitted by the fecal–oral route. The disease primarily affects children and young adults [3]. Typhoid and paratyphoid fevers, which are clinically identical multisystemic febrile illnesses, are caused by *Salmonella enterica* serotypes Typhi (*S.* Typhi) and Paratyphi (*S.* Paratyphi A, B, and C). Enteric fever affects over 13 million individuals and results in more than 110,000 deaths annually worldwide [4].

Almost 30% of community-acquired bacterial bloodstream infections in Asia and 10% in Africa are caused by *S*. Typhi [5,6]. On the other hand, *S.* Paratyphi A is responsible for up to 35% of all enteric fever episodes in India and Nepal, and over 60% in China [7,8]. The effectiveness of classical antimicrobial therapy is being undermined by the increasing resistance of *S.* Paratyphi A strains against multiple antibiotics [9]. Patients treated with ineffective antimicrobials show a poor clinical response and a high rate of complications and deaths. This is accompanied by prolonged fecal shedding, which sustains transmission and induces secondary cases [10,11].

In the last few years, several Vi-conjugate vaccines have become available for the prevention of enteric fever caused by *S.* Typhi [12,13,14,15] and have been recommended for use in infants [16]. Despite the existence of vaccines to prevent enteric fever caused by *S.* Typhi, there is currently no licensed vaccine against *S.* Paratyphi A [16]. In areas with a high incidence of paratyphoid fever, there is a risk that even a highly effective *S.* Typhi vaccine will be insufficient to reduce the burden of enteric fever as a whole, because of the increasing detection rates of *S.* Paratyphi A and the risk of serotype replacement [17].

Therefore, several groups have started working to develop bivalent vaccines to prevent disease caused by both *S*. Typhi and *S.* Paratyphi A [18].

The GSK Vaccines Institute for Global Health, in collaboration with Biological E, has combined the licensed Vi-CRM_197_ conjugate [19,20] with a new glycoconjugate constituted by *S.* Paratyphi A-specific O-antigen O:2 and CRM_197_ [21]. This new bivalent vaccine has recently completed a Phase 1 clinical trial in Europe (NCT05613205).

Prior to conducting clinical studies, the O:2-CRM_197_ conjugate was demonstrated to be immunogenic in animals and able to induce serum bactericidal activity against an *S*. Paratyphi A homologous strain [21].

The *S*. Paratyphi A O:2 repeating unit (RU) is composed of a trisaccharide backbone of rhamnose (Rha), mannose (Man), and galactose (Gal), with a terminal paratose (Par) at C-3 of Man, which confers the O:2 serospecificity. The O:2 also shows further decorations: a terminal glucose (Glc) can be linked to the C-6 of Gal, while the Rha residue can be variously substituted on C-2 and on C-3 with O-acetyl groups [22]. These O-antigen (OAg) modifications can be relevant immunological determinants [23].

In this study, we explored the OAg variations and their immunological effects on invasive clinical isolates coming from endemic regions of Nepal [24]. The O-antigens extracted from these strains were purified and fully characterized, showing differences in O:2 density, O-acetylation, and glucosylation levels.

To evaluate the relevance of these modifications, we investigated the ability of animal sera raised against O:2-CRM_197_ glycoconjugates to bind and kill the isolates. Sera generated in rabbits by O:2-CRM_197_ conjugates, formulated with or without adjuvant, and containing O:2 with different O-acetylation levels, were tested against these strains by flow cytometry and luminescence-based serum bactericidal assay (L-SBA) [25].

## 2. Materials and Methods

### 2.1. Bacterial Strains

The *Salmonella* Paratyphi A strains used in this study were invasive clinical isolates from Nepal and the commercial laboratory strain ATCC9150 (Table 1).

The Nepalese isolates were part of a larger panel of isolates from the blood cultures of patients and from chronic carriers in an urban hospital in Katmandu between 2005 and 2014 [24,26]. The entire panel of isolates was previously analyzed by whole-genome sequencing [26].

The strain identified as NVGH308 is the clinical isolate ED199, originated in 2006 from a patient with enteric fever in Katmandu [27].

All strains were stored at −80 °C in 20% glycerol stocks until use.

### 2.2. Conjugates Formulation

The O:2 OAg employed for the glycoconjugate synthesis was extracted from the *S*. Paratyphi A strain NVGH308 containing a Δ*tolR* mutation. The OAg extracted from this modified strain has a bimodal molecular weight (MW) population (two peaks, respectively, at 16 and 100 kDa), a 60.0% O-acetylation level, and a 79.0% glucosylation level [21].

By treating O:2 with NH_4_OH 1M, a completely not O-acetylated O:2 was obtained. Therefore, two different O:2-CRM_197_ glycoconjugates were synthesized by conjugating the CRM_197_ carrier protein with either the O-acetylated or the not O-acetylated OAg.

Formulations n. 1 and 2 (Table 2) were prepared by adding the glycoconjugates to the following excipients: water, Tris 25 mM pH 7.2, NaCl 9 g/L, and Alhydrogel (for a final concentration of Al^3+^ 0.75 mg/mL).

The synthesis of conjugates, the preparation of formulations 1 and 2, and their characterization are fully described in [21].

In formulation n. 3, the glycoconjugate used was the same O-acetylated O:2-CRM_197_ as in formulation n. 1, but in this case the Tris buffer was substituted by a phosphate buffer (pH 7.4) to prevent the adsorption of the conjugate on Alhydrogel. The adsorption of the conjugates to Alhydrogel was quantified by microBCA assay.

Table 2 summarizes the main characteristics of the formulations tested in this study.

### 2.3. Animal Studies

All animal studies were performed at Charles River Laboratories (France).

Groups of 8 New Zealand White rabbits (female, at least 2.5 kg in weight) were vaccinated intramuscularly with a 500 μL dose containing 25 µg of O:2-CRM_197_ glycoconjugates on study days 0 and 28. Equal amounts of sera collected 14 days post second vaccination from each group were pooled and used in this study. In Alfini et al., individual sera from rabbits immunized with O:2-CRM_197_–55.6% OAc were tested by ELISA and L-SBA against the strain NVGH308.

Body weight and temperature were monitored following vaccination for 72 h (body weight), or 24 h (temperature), or until the original values were restored in case body weight loss was >10% and temperature > 40 °C. Animal health conditions were monitored throughout all the studies, and no clinical signs of concern were reported.

### 2.4. Ethics and 3R Statement

All animal experiments were performed in accordance with the European Directive for the Use of Animals for Scientific Purposes 2010/63 and GSK policy on the Care, Welfare and Treatment of Animals. GSK is committed to the replacement, reduction, and refinement of animal studies (3Rs). Non animal models and alternative technologies are part of our strategy and are employed where possible. When animals are required, the application of robust study design principles and peer review minimizes animal use, reduces harm, and improves the benefit to studies.

### 2.5. O-Antigen Extraction and Characterization

**Scheme 1:**
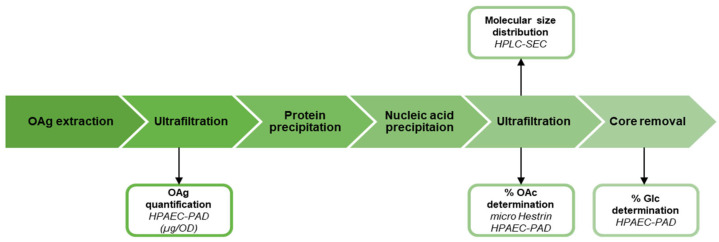
Step-by-step process of extraction, purification, and analytical characterization of O-antigens from *S.* Paratyphi A strains.

O-antigens were extracted from the bacterial strains listed in Table 1 following a procedure previously reported [28] with few modifications (Scheme 1).

A daytime culture was started from frozen stock in 5 mL of Luria–Bertani (LB) for 6–8 h at 37 °C and 180 rpm prior to being transferred to a 50 mL overnight culture (16–18 h) in LB at 37 °C and 180 rpm. Afterwards, the bacterial culture was centrifuged (5000 rcf, 30 min), the supernatant discarded, and the pellet was resuspended in acetic acid 2% (*v/v*) and incubated at 100 °C for 5 h. Through a second centrifugation step (5000 rcf, 45 min), the OAg solution was recovered and separated from the pellet that contains lipid A, membrane debris, and proteins. After the hydrolysis, the pH was increased to 6 by adding 28% NH_4_OH. The solution was then ultrafiltered with Amicon Ultra Centrifugal Filter 10 kDa (Merck, Darmstadt, Germany) to remove the lower molecular mass impurities, first against NaCl 1M (3 cycles, 1895 rcf, 20 min) and then against water (3 cycles, 1895 rcf, 20 min). The OAg was then quantified by High-Performance Anion-Exchange Chromatography coupled to a Pulsed Amperometric Detector (HPAEC-PAD) [29].

The ultrafiltered solution was then further purified by adding citrate buffer pH 2.7 (final concentration 20 mM) to precipitate protein impurities. The supernatant was further purified by adding Na_2_HPO_4_, EtOH, and CaCl_2_ to have 18 mM NaH_2_PO_4_, 24% EtOH (*v/v*), and 200 mM CaCl_2_ in the final mixture to co-precipitate nucleic acid. The solution containing OAg was finally ultrafiltered in Amicon Ultra 10 kDa vs. NaCl 1M (3 cycles, 1895 rcf, 20 min) and vs. water (3 cycles, 1895 rcf, 20 min). The resulting solution was used to calculate the OAg molecular weight with GPC using a Dextrans calibration curve, as previously reported [21], and the O-acetylation level.

The O-acetyl ester content was determined by performing a modified microplate procedure of the Hestrin assay [21].

Finally, the lipid A core was removed by ultrafiltration using Amicon Ultra with a higher cut-off (30 kDa) and the OAg glucose content was estimated by HPAEC-PAD analysis and reported as the molar ratio to mannose.

### 2.6. Flow Cytometry Binding Assay

Overnight cultures of strains, listed in Table 1, were started from frozen stocks and then diluted in 7 mL of warm LB medium from an optical density at OD_600nm_ =0.05 and incubated at 37 °C with 180 rpm agitation in an orbital shaker until they reached an OD_600nm_ between 0.18 and 0.25. Then, 1 mL of working culture was pelleted at 12,000× *g* for 10 min and resuspended in 1 mL PBS + 1% BSA buffer. An amount ot 50 mL of bacteria suspension was then added to a 96-well plate (Costar), washed with 100 mL PBS + 1% BSA, centrifuged at 4000× *g* for 10 min, and the supernatant was discarded. The bacteria in the pellet were fixed by resuspending each well in 50 µL Cytofix (BD) solution. After incubation for 30 min at 4 °C, the plate was centrifuged at 4000× *g* for 10 min, the supernatants were discarded, and the bacterial pellets were washed twice with PBS + 1% BSA buffer. Then, the bacteria pellets were incubated with primary antibodies (pooled sera from rabbits obtained by immunizing with the constructs reported in Table 2 and diluted to 1:500 in PBS + 1% BSA; pre-immune sera diluted to 1:500 in PBS + 1% BSA were used as negative controls) for 30 min under orbital shaking at 750 rpm at 25 °C. The bacterial pellet was washed twice with 100 mL PBS + 1% BSA buffer and then incubated with 50 mL of anti-rabbit IgG-PE (BD) for 30 min with shaking at 750 rpm at 25 °C. The bacterial pellets were washed twice with 100 µL PBS + 1% BSA buffer and resuspended in 150 mL PBS + 1% BSA buffer. The plate was acquired with BD Accuri flow cytometer (BD, Franklin Lakes, U.S.) with CSampler Plus. A gating was first set in the forward (FSC) and side scatter (SSC). The acquisition was set to 10,000 bacterial events inside the bacterial gating, with a slow acquisition speed, and a 4500-threshold set for FSC-H. The samples were analyzed in FlowJo. Bacterial cells were gated manually leaving out agglutinated bacteria or clumps and the geometric mean of fluorescence intensity (MFI) was extrapolated.

### 2.7. Luminescence-Based Serum Bactericidal Assay

The luminescence-based serum bactericidal assay (L-SBA) was performed as previously described [25]. Briefly, a daytime culture was started at OD_600nm_ = 0.05 from an overnight culture, until it reached an OD_600nm_ between 0.22 and 0.25. The bacteria were then diluted at 1:600 and sera were heat-inactivated (HI) at 56 °C for 30 min to remove endogenous complement activity.

To estimate the signal released from the initial concentration of bacteria, the luminescence at T0 was measured. The reaction mixtures were prepared in a final volume of 100 µL, containing 10 µL of the bacterial cells diluted, 25 µL of baby rabbit complement (Cederlane) as an external source of complement, and 25 µL of HI serum. The plates were incubated for 3 h at 37 °C and then centrifuged at 25 °C for 10 min at 4000× *g*. The supernatant was discarded to remove the ATP derived from dead bacteria and L-SBA reagents, and the remaining live bacterial pellets were resuspended in 100 µL PBS. The luminescent signal was detected by testing the bacterial pellet mixed 1:1 with BacTiter-Glo Reagent (Promega, Madison, U.S.) using a Synergy luminometer (Biotek, Winooski, U.S). Bactericidal serum titers were calculated by fitting a four-parameter logistic curve to the raw luminescence signal in function to the different Log sera dilutions by GraphPad Prism 9.3.1. Bactericidal titers were represented by the reciprocal serum dilution necessary to obtain 50% bacterial growth inhibition.

## 3. Results

### 3.1. S. Paratyphi A Isolates Express OAg with Different Levels of O-Acetylation, Glucosylation, and Molecular Weight

O-antigens were isolated from the *S.* Paratyphi A strains, and fully characterized in terms of polysaccharide chain length, quantity, percentage of O-acetyl groups, and Glc content (see Table 1).

The bacteria were grown in the same conditions and reached a similar OD600 (range from 3.2 to 4.2). Despite that, the OAg amount produced by the strains was different, ranging from 44 µg of polysaccharide per OD for the laboratory strain ATCC9150 to 396 µg of polysaccharide per OD for the clinical isolate 02TY046.

**Figure 1:**
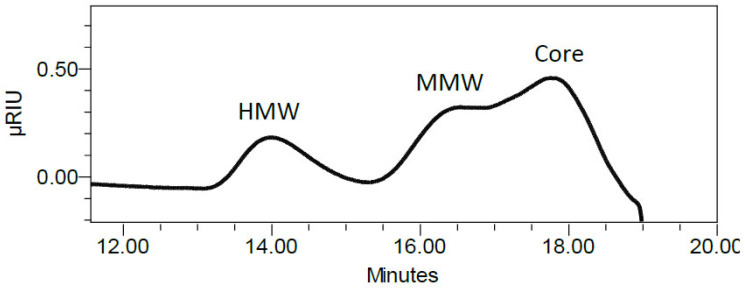
HPLC-SEC with the differential refractive index detection profile of ED705 OAg, as a representative isolate. The profile shows the HMW population’s peaks and peaks corresponding to the MMW population and core, which are not completely resolved.

Most isolates presented OAg populations at two different molecular weights: a high molecular weight population (HMW) at around 90 kDa, and a medium molecular weight (MMW) population at around 10 kDa, whose peak is not completely separated from the peak relative to the core, or the core plus a few RUs only at around 2 kDa (Figure 1). The OAg extracted from isolate 02Y004 shows the peak corresponding to the HMW population slightly above the average, at 111.3 kDa, while isolates 02TY046 and NVGH308 did not present the MMW peak (differences with the strain NVGH308 Δ*tolR* used for the glycoconjugate synthesis are probably related to the different growth conditions).

None of the strains presented completely the O-acetylated O:2 and only strain ED766 presented OAg with 100% O-acetylation. All other isolates showed a range of O-acetylation levels, with the laboratory strain ATCC9150 characterized by the lowest O-acetylation level (17%) of the panel.

**Figure 2:**
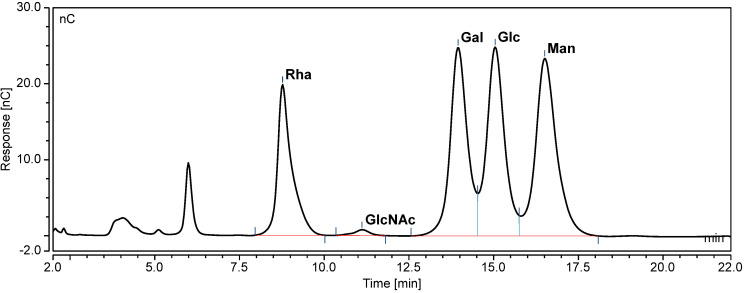
The HPAEC−PAD profile of the OAg 01TY544 hydrolyzed after the core’s removal, as a representative isolate. The monomers constituting the OAg RU are highlighted: Rha, Gal, Glc, and Man. The calculated Glc/Man nmol ratio gives the glucosylation level of the OAg. The low peak at a retention time of 11.2 min is the glucosamine (GlcNAc) residual coming from the core.

Glucosylation levels, calculated on the OAg hydrolyzed after the core’s removal, were in the range of 49 to 95% (Figure 2).

### 3.2. Sera Raised Against O:2 CRM_197_ Conjugates Are Able to Bind and Kill Clinical Isolates with Different OAg Characteristics

**Figure 3:**
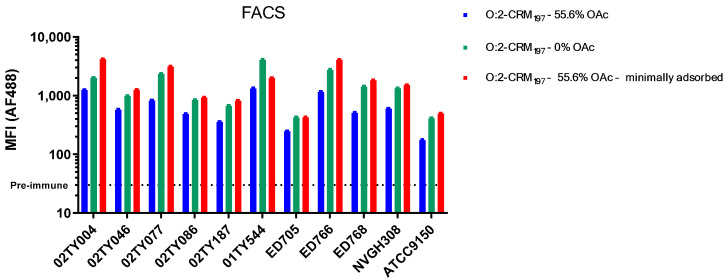
Individual MFI values for all strains tested in flow cytometry binding assay using sera generated in rabbits with the three O:2 conjugates (Table 2) differing for the O-Acetylation level and the adsorption on Alhydrogel.

Rabbit sera obtained after immunization with 55.6% O-acetylated O:2-CRM_197_ vaccine candidate (Table 2, formulation n. 1) were able to bind all clinical isolates by flow cytometry (Figure 3).

**Figure 4:**
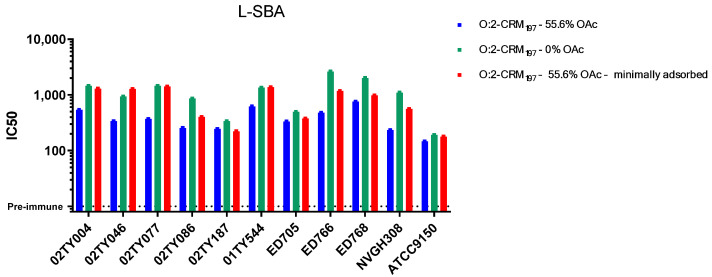
Individual IC50 for all strains tested in L-SBA using sera generated in rabbits with O:2 conjugates differing for the O-Acetylation level, and with adsorbed or minimally adsorbed O-acetylated conjugate.

Complement is a key factor in each L-SBA assay and the appropriateness of the source and amount must be evaluated; therefore we first examined the susceptibility of bacterial strains to complement. All the strains, independently of their OAg characteristics, presented analogous sensitivity to non-specific complement killing and were tested by L-SBA using the same conditions and same amount of exogenous complement. Rabbit sera killed all tested clinical isolates (Figure 4), regardless of their different OAg characteristics.

To verify if the O:2-CRM_197_ O-acetylation level plays a role in the bactericidal activity against the differently O-acetylated isolates, we tested sera after immunization with the not O-acetylated conjugate (Table 2, formulation n. 2). The sera were still able to bind (Figure 3) and kill (Figure 4) all the isolates, regardless their O-acetylation levels.

Also, we wanted to investigate the impact of adsorption on the Alhydrogel of the glycoconjugates. Comparing sera coming from immunizations with either adsorbed or minimally adsorbed O:2-CRM_197_ (Table 2, formulations n. 1 and 3), we found a similar binding and bactericidal activity against the tested strains (Figure 3 and Figure 4).

## 4. Discussion

Typhoid and paratyphoid fever still represent a major global health problem, particularly in children in South and South-East Asia. Despite the successful introduction of vaccines against *Salmonella* Typhi, there is still no vaccine against *S.* Paratyphi A, and *S.* Paratyphi A-containing vaccines have been identified as a strategic priority by the WHO [30].

We have previously shown that animal sera obtained after O:2-CRM_197_ immunization can effectively kill an *S*. Paratyphi A strain whose OAg was used to generate the O:2-CRM_197_ candidate vaccine [21]. However, *S.* Paratyphi A strains may have O-antigens with different levels of O-acetylation, glucosylation, and relative expression of OAg.

Therefore, we tested a panel of endemic strains to assess possible differences in their OAg characteristics, and to evaluate if our *S.* Paratyphi A vaccine candidate could generate an antibody response able to neutralize them. A vaccine effective across multiple strains would minimize the risk of encountering strains able to escape its potential coverage [31].

Even though some work has been done to study the genetic variation in *S*. Paratyphi A isolates [32] and their population structure in specific endemic regions [26], very little is known about variation in OAg features among clinical isolates. Genomic studies have found a relative homogeneous population structure, where genotype replacement may be driven by reduced susceptibility to antibiotics (i.e., fluoroquinolones) and by possible changes in virulence factors, providing an increased fitness.

A more recent study from the University of Cambridge provided a deeper understanding of the phenotypic variations in a collection of *S*. Paratyphi A OAg isolates from Nepal, identifying two specific variations in the LPS, “typical” and “variant”, that could be related to some structural modifications of the OAg [24].

In this report, for the first time, we chemically characterized the OAg from a representative panel of clinical isolates, and included the laboratory strain ATCC9150, whose genome was the first to be sequenced in 2004 [33]. Excluding the less relevant laboratory strain, we found that, in the same growth conditions, *S*. Paratyphi A isolates presented an amount of OAg/OD production with a 6-fold difference between the highest and the lowest producer; OAc levels with a 3-fold difference between the highest and the lowest O-acetylated strain (mean around 73% O-acetylation); and glucosylation levels with a 2-fold difference between the highest and the lowest glycosylated strain (mean around 72%). Most strains presented OAg with a bimodal MW distribution at medium and high MW.

This variation in OAg characteristics is of high interest and, for other *S. enterica* serovars such as *S*. Typhimurium [34], may be indicative of different selective pressures.

The OAg moiety of lipopolysaccharides is considered a possible target of protective immune responses, and OAg-based vaccine candidates are in development for additional *Salmonella* serovars like Typhimurium and Enteritidis [35], along with other *Enterobacteriaceae* pathogens like *Shigella* spp [36], *K. pneumoniae*, etc. [37]. For *Salmonella* serovars, OAg characteristics such as molecular size and O-acetylation levels and position have been shown to play an important role in virulence [38,39] and in the ability of a OAg-based candidate to induce immunogenic responses in animals [40]. More specifically, O-acetyls were confirmed to be immunodominant epitopes of *S*. Typhimurium OAg [41] and, also for *S*. Typhi, O-acetylation was found to be a critical determinant of immunogenicity of the Vi polysaccharide antigen [42,43,44].

In the case of *S*. Paratyphi A, OAg MW may impact the immunogenicity of O:2-conjugates: Alfini et al. [21] showed higher immune responses in mice (but not in rabbits) for O:2-CRM_197_ with conjugates containing O:2 at a low MW, while Konadu et al. showed the opposite for a different O:2-TT conjugate [23]. In addition, O-acetylation may be an important determinant of functional humoral response and both Alfini et al. and Konadu et al. showed the enhanced immunogenicity and bactericidal activity of mouse sera from conjugates containing O-acetylated OAg. Clearly, the ability of OAg features to influence the response to a glycoconjugate should be evaluated concomitantly with multiple other parameters, and, most importantly, in relation to the animal species used.

In this report, using rabbit as animal model, we found that sera from de O-acetylated O:2-CRM_197_ conjugates were as able to bind and kill the *S*. Paratyphi A strains as sera from O-acetylated conjugates. This finding suggests a non-critical role in the bactericidal activity of rabbit antibodies against O-acetylated epitopes and it differs from what had previously been found in mice [21].

Similarly, adsorption to Alhydrogel did not seem to contribute to enhance the conjugates immunogenicity.

That said, we observed a trend for the higher binding and increased bactericidal activity of sera raised by minimally adsorbed and not O-acetylated OAg-conjugates. The significance of this finding is currently unknown, and it is similarly unknown what animal species can best predict the response in humans.

Clearly, most relevant results will come from the recently completed Phase 1 clinical study, where a bivalent conjugate vaccine containing TCV and O:2-CRM_197_, adsorbed or not to Alhydrogel, has been administered to healthy adults.

## 5. Conclusions

In conclusion, the results of this work show that our O:2-CRM_197_ conjugate could theoretically protect against multiple clinical isolates, regardless of their OAg characteristics, thus supporting the further development of the bivalent vaccine against Typhi and Paratyphi A. These results will be confirmed with the most relevant clinical sera, as soon as available.

## Figures and Tables

**Table 1:** Clinical isolates of *Salmonella* Paratyphi A strains and their OAg analytical characterization including OAg production, size, O-acetylation, and glucosylation levels.

Isolate ID	OAg µg/OD	Size (kDa)		%OAc *	%Glc *
		HMW	MMW; Core		
02TY004	307	111.3	11.4; 3.1	90	64
02TY046	396	94.4	2.4	61	76
02TY077	255	98.5	10.1; 2.6	75	88
02TY086	263	84.3	11.1; 2.5	94	51
02TY187	195	85.0	11.9; 2.5	54	51
01TY544	273	96.3	13.0; 3.2	87	89
ED705	65	975	8.6; 2.7	73	49
ED766	139	91.6	10.4; 2.7	100	79
ED768	245	92.0	11.0; 2.5	37	95
ATCC9150	44	99.7	6.6; 2.6	17	57
NVGH308	219	86.1	2.5	61	78

* % OAc and % Glc are expressed as molar ratio % with respect to RU.

**Table 2:** An overview of the three different O:2-CRM_197_ formulations synthetized in the Alfini et al. study [21], differing by O-acetylation level and adsorption on Alhydrogel.

Entry *n*	Conjugate Formulation	O:2 O-Acetylation	Conjugate Adsorption on Alhydrogel	Osmolality mOsm/kg	pH
1	O:2-CRM_197_–55.6% OAc	55.6%	98.1%	278	7.76
2	O:2-CRM_197_–not OAc	0%	99.0%	298	7.45
3	O:2-CRM_197_–55.6% OAc–minimally adsorbed	55.6%	14.1%	282	7.67

## Data Availability

The original contributions presented in this study are included in the article. Further inquiries can be directed to the corresponding author(s).
